# Effect of Al Content in the Mg-Based Alloys on the Composition and Corrosion Resistance of Composite Hydroxide Films Formed by Steam Coating

**DOI:** 10.3390/ma12071188

**Published:** 2019-04-11

**Authors:** Takahiro Ishizaki, Tomohiro Miyashita, Momo Inamura, Yuma Nagashima, Ai Serizawa

**Affiliations:** 1 Department of Materials Science and Engineering, College of Engineering, Shibaura Institute of Technology, Tokyo 135-8548, Japan; serizawa@shibaura-it.ac.jp; 2 Materials Science and Engineering, Graduate School of Engineering and Science, Shibaura Institute of Technology, Tokyo 135-8548, Japan; mb18030@shibaura-it.ac.jp (T.M.); mb18002@shibaura-it.ac.jp (M.I.); AC15052@shibaura-it.ac.jp (Y.N.)

**Keywords:** magnesium alloys, steam coating, composite hydroxide film, corrosion resistance

## Abstract

Mg alloys are expected to be used in fields of the transportation industry because of their lightweight property, however, they show low corrosion resistance. To improve the corrosion resistance, preparation of the protective film on Mg alloys is essential. In this study, composite hydroxide films were prepared on three types of Mg alloys with different aluminum contents—that is, AZ31, AZ61, and AZ91D—by steam coating to investigate the relationship between the Mg-Al layered double hydroxide (LDH) content in the film and the Al content in the Mg alloys. Scanning electron microscopy (SEM) observation demonstrated that films were formed densely on all Mg alloy surfaces. X-ray diffraction (XRD) analyses revealed that all films prepared on AZ61 and AZ91D were composed of Mg(OH)_2_, AlOOH, and Mg-Al LDH, while the film containing Mg(OH)_2_ and Mg-Al LDH were formed only on AZ31. The Mg-Al LDH content in the film prepared on AZ61 was relatively higher than those prepared on AZ31 and AZ91D. The content of AlOOH in the film increased with an increase in the Al content in the Mg alloys. The film thickness changed depending on the treatment time and type of Mg alloy. Polarization curve measurements in 5 mass% NaCl solution demonstrated that the film prepared on the AZ61 showed complete passive behavior within the potential range of −1.0 to −0.64 V. In addition, immersion tests in 5 mass% NaCl aqueous solution for 480 h demonstrated that the film on the AZ61 had superior durability against 5 mass% NaCl aqueous solution. These results indicated that the film on the AZ61 had the most superior corrosion resistance among all samples. The results obtained in this study suggest that the LDH content in the film could be related to the corrosion resistance of the film.

## 1. Introduction

Magnesium alloys have excellent physical and mechanical properties, such as good electromagnetic shielding and a high strength-to-weight ratio. Especially, the lightweight property has attracted much attention because Mg has a low density of 1.74 g cm^−3^, which is one-quarter of iron and two-thirds of aluminum. A reduction in the weight of materials is highly desirable in the field of electronics and the transportation industry [1,2,3]. Mg and its alloys are one of the candidate materials for this purpose. However, Mg alloys are an electrochemically active and base metal, so they tend to suffer severe corrosion [4,5]. Thus, the preparation of a protective film on Mg and its alloys is required to improve the corrosion resistance. Chemical conversion treatment and anodic oxidation are representative and conventional surface treatment methods for Mg and its alloys because of their low cost and easy operation [6,7,8,9,10,11]. Chromate system chemical conversions are well known as being the most famous and effective chemical conversion process to prepare a protective film on various metal surfaces [12,13,14]. However, these treatments have disadvantage, such as the usage of harmful chemicals containing hexavalent chromium (Cr^6+^). The chemical conversion film prepared using Cr^6+^ showed high corrosion resistance and self-repairing capability. However, the usage of this chemical has been strictly prohibited by many countries because of high toxic compounds, such as irritants, and is caustic to skin and mucous membranes [15]. Therefore, it is necessary to develop environmentally friendly Cr^6+^-free surface treatment methods [16,17,18]. Some new environmentally friendly chemical conversion treatments, such as stannate, rare earth salt, and phosphate/permanganates conversion treatments, have been developed [19,20,21,22,23,24]. However, the corrosion resistance of the films prepared by these treatments has not been adequate for use as structural materials toward transportation, such as automobiles, railways, and so on, because the film thickness was thin. Therefore, the development of a novel surface treatment to impart high corrosion resistance is highly desirable. We proposed a novel and environmentally friendly coating method, that is, steam coating, for AZ31 Mg alloy [25,26]. Steam coating is a chemical free method and only pure water is used as a steam source [25]. In addition, the steam coating for Mg alloys enables the formation of a high corrosion resistant film [27,28]. The films grow directly from the substrate surface by a chemical reaction with water, leading to excellent adhesion between the film and substrate. The films formed on AZ31 are composed of Mg(OH)_2_ and Mg-Al layered double hydroxide (Mg-Al LDH: Mg_1−*x*_Al*_x_*(OH)_2_(CO_3_)*_x_*_/2_·*n*H_2_O). 

LDHs have layered structures composed of positively charged sheets and negatively charged ions. The negative ions, such as CO_3_^2−^ and NO_3_^−^, are intercalated between the positively charged sheets to compensate the charge balance of the LDH. It has been reported that LDHs were effective at improving the corrosion resistance because they allowed anion exchangeability between intercalated anions and corrosive ions, thus functioning as a corrosion retardation film [29,30,31]. Lin et al. demonstrated that carbonate Mg-Al LDH could exchange the intercalated CO_3_^2−^ into Cl^−^ anion in a corrosive environment, resulting in a protective layer of the Mg alloy against corrosion [32]. Tedim et al. also reported that Zn-Al LDH intercalated with nitrate anions could trap Cl^−^ in a corrosive environment into the interlayer of the LDH and the addition of the LDH to a polymer layer considerably reduced the permeability of corrosive chloride anions through the protective coatings [33]. Thus, the increase in the LDH content in the film could improve the corrosion resistance. 

In the steam coating process, films composed of Mg(OH)_2_ and Mg-Al LDH were formed through the reaction of Mg alloy with water, and the Al sources to form Mg-Al LDH could be supplied from the Mg alloys. Thus, the LDH content in the film can be considered as being dependent on the Al content in the Mg alloys. However, the relationship between the LDH content in the film and the Al content in the Mg alloys has not yet been investigated.

In this study, we aimed to investigate the relationship between the LDH content in films prepared by steam coating and the Al content in Mg alloys by using three types of Mg alloys with different Al contents, i.e., AZ31, AZ61, and AZ91D. The resulting samples were characterized using XRD, field emission scanning electron microscopy (FESEM), and field emission scanning electron microscopy-energy dispersive spectroscopy (FESEM-EDS). In addition, the correlation between the corrosion resistance of the films and the LDH content in the films were also examined. The corrosion resistance of the three types of Mg alloys covered with films with different LDH contents was investigated by potentiodynamic polarization curve measurements in 5 mass% NaCl aqueous solution. 

## 2. Materials and Methods 

AZ31 (composition: 3 mass% Al, 1 mass% Zn, 0.3 mass% Mn, and balance Mg), AZ61 (nominal composition: 6 mass% Al, 1 mass% Zn, 0.3 mass% Mn, and balance Mg), and die cast AZ91D (nominal composition: 9 mass% Al, 1 mass% Zn, 0.3 mass% Mn, and balance Mg) Mg alloys were purchased from standard-testpiece co. and were used as substrates. AZ31 and AZ91D samples were cut in the form of plates with the dimensions of 20 mm × 20 mm × 2 mm and AZ61 sample was cut in the form of plates with the dimensions of 20 mm × 20 mm × 1 mm. All substrates were grinded with SiC paper up to #1200 grit, and then ultrasonically cleaned in ethanol before use. The cleaned substrates were introduced into Teflon-lined autoclave. Twenty mL of pure water was located at the bottom of the autoclave as a steam source. The autoclave was heated at a temperature of 423 K for 3 to 7 h.

The surface morphologies and cross sections of the film coated samples were observed by a field emission scanning electron microscopy (FESEM, JSM-IT300HR, JEOL, Tokyo, Japan). The atomic composition was determined using FESEM-EDS (FESEM, JSM-IT300HR, JEOL, Tokyo, Japan). The crystal structure of the samples was identified by X-ray diffraction (XRD, Ultima IV, Rigaku, Tokyo, Japan) at a glancing angle of 1°. The XRD data were recorded on a powder diffractometer with CuKα radiation (λ = 0.15418 nm) operated (40 kV, 40 mA) within the range of 5° to 80° at a scanning rate of 2θ = 4° min^−1^. 

The corrosion resistant performance was examined using electrochemical measurements and immersion tests in 5 mass% NaCl aqueous solution. All electrochemical measurements were performed in 5 mass% NaCl aqueous solution (pH = 6.5), at room temperature, using a computer-controlled potentiostat (Princeton Applied Research, VersaSTAT3, Ametek, Berwyn, PA, USA) under open circuit conditions. The Mg alloy substrates covered with and without film and a platinum plate were used as working and counter electrodes, respectively. Silver/silver saturated-chloride (Ag/AgCl) was used as a reference electrode. Hereafter, all potentials in this study are described as potentials versus Ag/AgCl. Magnesium alloy substrate was immersed in the NaCl aqueous solution for 30 min and potentiodynamic polarization curves were subsequently measured at a scanning rate of 0.5 mV s^−1^ from −100 to +800 mV versus the open circuit potential (OCP). The values of the corrosion potential (E_corr_) and corrosion current density (i_corr_) were derived from the potentiodynamic polarization curves by Tafel extrapolation using the electrochemical software, CorrView (CorrView International). The composite hydroxide film coated Mg alloys were immersed in 5.0 mass% NaCl solution (20 mL) at 308 K for up to 480 h for the immersion test to investigate the durability of the films prepared on AZ31, AZ61, and AZ91D Mg alloys. The substrates with a dimension of 20 mm × 20 mm × 3 mm were applied for all immersion tests.

## 3. Results and Discussion

### 3.1. Surface Characterizations

#### 3.1.1. Surface Morphologies

**Figure 1:**
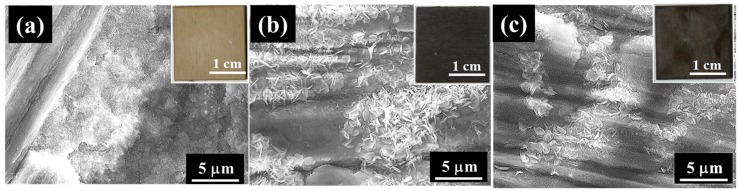
SEM images of films prepared at 423 K for 5 h on (**a**) AZ31, (**b**) AZ61, and (**c**) AZ91D. All insets show the appearance of the film surfaces.

Figure 1 shows SEM images of the film prepared at 423 K for 5 h on (a) AZ31, (b) AZ61, and (c) AZ91D. All insets show the appearance of the film surfaces. After steam coating, films were formed densely on all Mg alloy surfaces. The film prepared on AZ31 had compact and rough regions on the film surface. The AZ31 surface appeared to be covered uniformly with the film from the appearance of the film on AZ31. On the film prepared on AZ61, many plate-like structures inclined to the substrate surface and a relatively compact surface could be observed. The AZ61 surface also appeared to be covered uniformly with the film from the appearance of the film on AZ61. Although the film surface on AZ91D was relatively flat, needle- and plate-like structures with sizes of less than 1 µm could be seen locally. The appearance of the film on AZ91D confirmed the formation of the uniform film. From these SEM images, it was confirmed that all Mg alloy surfaces were covered with film.

#### 3.1.2. FESEM-EDS Analyses of the Film Surfaces

**Figure 2:**
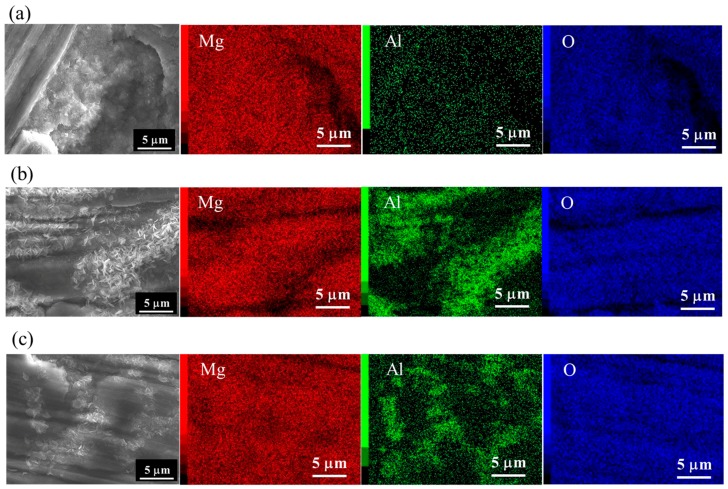
FESEM and elemental Mg, Al, and O mapping images of film prepared at 423 K for 5 h on (**a**) AZ31, (**b**) AZ61, and (**c**) AZ91D.

FESEM and elemental Mg, Al, and O mapping images of films prepared at 423 K for 5 h on (a) AZ31, (b) AZ61, and (c) AZ91D are shown in Figure 2. SEM images shown in Figure 2 are the same regions as those of Figure 1. The detected elements were C, O, Mg, and Al for all films. The elemental C may be attributed to surface contamination and MgCO_3_ formed on the surface. After steam coating, the atomic concentration of O increased and those of Mg and Al decreased. The composition ratio of Mg to O for all films was estimated to be approximately 0.5, independent of the types of Mg alloys. This indicates that all films contain Mg(OH)_2_. Ishizaki et al. reported that films prepared at 423 K on AZ31 by steam coating were composed of Mg(OH)_2_ and Mg-Al LDH [25]. FESEM-EDS analyses demonstrated that the Al atom was concentrated locally at the existence positions of the needle- and plate-like structures on the films formed on AZ61 and AZ91D as shown in Figure 2b,c, indicating that needle- and plate-like structures can be considered to be formed as Al-containing compounds. On the other hand, little Al trace was detected on the films prepared on AZ31 as shown in Figure 2a.

### 3.2. Crystal Phases of Films Prepared on AZ31, AZ61, and AZ91D

**Figure 3:**
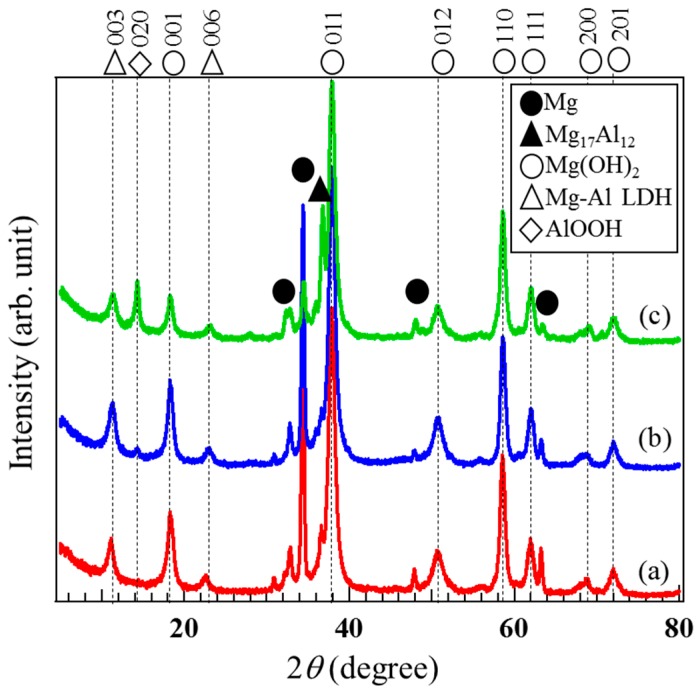
XRD patterns of the films prepared at 423 K for 5 h on each Mg alloy: (a) AZ31, (b) AZ61, and (c) AZ91D.

To identify the crystal phases formed in the films prepared on AZ31, AZ61, and AZ91D, XRD measurements were carried out. XRD patterns of the films prepared at 423 K for 5 h on (a) AZ31, (b) AZ61, and (c) AZ91D Mg alloys are shown in Figure 3. Several peaks at approximately 2θ = 18, 38, 51, 58, 62, 68, and 72° assigned to the 001, 011, 102, 110, 111, 200, and 201 reflections of brucite-type Mg(OH)_2_ can be detected. A peak observed at 2θ = 14° on the XRD patterns of the films on AZ61 and AZ91D is attributed to 020 reflection of bohmite-type AlOOH. In addition, two peaks at around 2θ = 11.6 and 23.5° assigned to the 003 and 006 reflections of the carbonate type Mg-Al layered double hydroxide (LDH) are clearly observed on all XRD patterns. From the XRD patterns, it was concluded that all films prepared on AZ61 and AZ91D are composed of a mixed structure of crystalline Mg(OH)_2_, AlOOH, and Mg-Al LDH phases whereas Mg(OH)_2_ and Mg-Al LDH is contained in the film prepared on AZ31. These results agree well with the results of the FESEM-EDS analyses.

To investigate the effect of the Al content in the Mg alloys on the crystal phases formed in the films prepared by steam coating, the ratio of crystal phases formed in the film was roughly estimated using the peak intensity ratio of 011 reflection of Mg(OH)_2_, *I*_Mg(OH)2-011_, at approximately 2θ = 38.0°, 003 reflection of Mg-Al LDH, *I*_LDH-003_, at approximately 2θ = 11.6°, and 020 reflection of AlOOH, I_AlOOH-020_, at approximately 2θ = 14.0° to total peak intensities; that is, *I*_Mg(OH)2__-001_ + *I*_LDH-003_ + *I*_AlOOH-020_, obtained from XRD patterns of the films prepared on (a) AZ31, (b) AZ61, and (c) AZ91D Mg alloys. The peak intensities for these reflections correspond to the strongest intensities shown in the standard powder diffraction data for Mg(OH)_2_, Mg-Al LDH, and AlOOH. For example, the ratio of the crystalline Mg-Al LDH phase in the film was calculated as follows; *I*_LDH_ ratio = *I*_LDH-003_/(*I*_Mg(OH)2-101_ + *I*_Mg(OH)2-001_ + *I*_AlOOH-020_). The calculated results are listed in Table 1. The film prepared on AZ31 had the highest Mg(OH)_2_ component ratio among all samples. The ratio of the crystalline Mg-Al LDH phase formed in the film prepared on AZ61 showed the highest value among all samples. The ratio of the crystalline AlOOH phase in the film prepared on AZ91D was higher than those prepared on AZ31 and AZ61. The ratio of the AlOOH phase increased with an increase in the Al content in the Mg alloys. These results agree well with the results of EDS analyses.

### 3.3. Film Thickness

**Figure 4:**
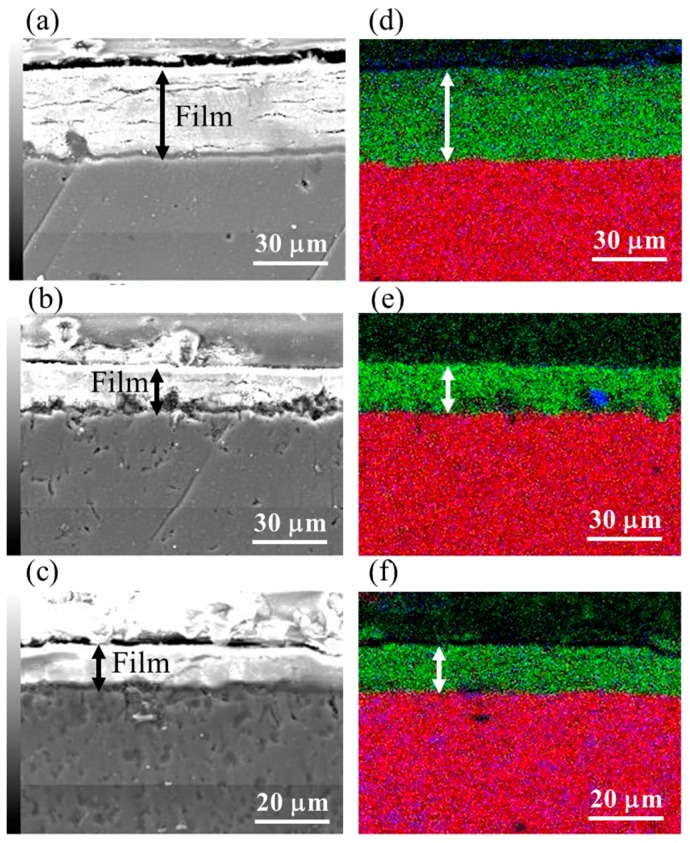
Cross sectional SEM and overlapped mapping images for Mg (red), Al (blue), and O (green) of film prepared at 423 K for 5 h on (**a**,**d**) AZ31, (**b**,**e**) AZ61, and (**c**,**f**) AZ91D.

Film thicknesses of the films prepared at 423 K for different treatment times on AZ31, AZ61, and AZ91D were estimated using cross-sectional SEM images and overlapped mapping images for Mg, Al, and O. The cross-sectional SEM images and overlapped mapping images for the Mg, Al, and O of the films prepared at 423 K for 5 h on AZ31, AZ61, and AZ91D are shown in Figure 4. The film thicknesses obtained from cross-sectional SEM images of the films prepared on AZ31, AZ61, and AZ91D Mg alloys at 423 K for 5 h were found to be 37.8, 20.0, and 11.9 µm, respectively. From the cross sectional FESEM images, although the films had several cracks parallel to the substrate surface, all films were relatively dense. The elemental mapping images in Figure 4 demonstrated that the film mainly included Mg and O, indicating that the film comprised mainly of Mg(OH)_2_. This result agrees well with the results of the XRD patterns. A little trace of Al can also be observed, evidencing that Mg-Al LDH and AlOOH exist locally in the film. The relationship between the film thickness and treatment time is listed in Table 2. The film thickness increased with an increase in the treatment time, independent of the type of Mg alloy. The difference in the thickness of the films prepared under the same conditions on different Mg alloys might be due to the difference in the existing amounts of the compound phases, such as the β-phase (Mg_17_Al_12_), in each Mg alloy because the existence of compound phases can disturb the formation of the Mg(OH)_2_ film, which is the main component in the film. It has been reported that the presence of the β-phase prevented Mg alloy from corroding [34]. Thus, the formation of the Mg(OH)_2_ film by the steam coating process could be hindered due to the presence of the β-phase. Thus, the film thickness could be decreased with an increase in the Al content of the Mg alloys. In addition, the increased ratio of the films was not ¥ proportional to the treatment time. This could be because a constant time of changing from the liquid to gas phases of the water introduced into the autoclave was required. Thus, the film growth rate could be slow at the initial stage of the steam process and gradually get faster with the progress in the phase transition of the water. After completing the phase transition of the water, the film growth rate could be kept almost constant.

During the steam coating process, after the phase transition of the water from the liquid to gas phases, Mg alloys can react with water vapor, leading to the formation of Mg(OH)_2_. The Mg(OH)_2_ was considered to be formed through the following chemical reaction (Equation (1)):

Mg + 2H_2_O → Mg(OH)_2_ + H_2_↑ (1)

In the case that MgO is formed as a surface oxide on the Mg alloy surface, the Mg(OH)_2_ can be formed via the following chemical reaction (Equation (2)):

MgO + H_2_O → Mg(OH)_2_ (2)

Consequently, the Mg(OH)_2_ was considered to be formed as a main crystal phase in the film prepared by steam coating. The Mg-Al LDH in the films could be considered to be formed via a solid phase reaction of formed Mg(OH)_2_ and Al atoms as a solid solution in Mg alloys [31]. The Al atoms might be incorporated into the Mg(OH)_2_ crystals by diffusion of the Al atoms. The incorporation of Al atoms into the Mg(OH)_2_ crystals led to the formation of positively charged layered structures. Thus, the positively charged layered structures could intercalate negatively charged substances, i.e., anions, between the layers to compensate the electric balance, resulting in the formation of Mg-Al LDH. The AlOOH in the films could be formed via a chemical reaction of the residual Al atoms and water vapor as the following reaction (Equation (3)) [35]:

2Al + 4H_2_O → 2AlOOH + 3H_2_ (3)

Although the formation of Al_2_O_3_ in the films can be considered, no peak attributable to the Al_2_O_3_ phase was detected in the XRD pattern as shown in Figure 3. It has been reported that the Al_2_O_3_ phase is, thermodynamically, the most stable at more than 753 K [36]. Serizawa et al. also demonstrated that only the crystal AlOOH phase was formed on Al alloys by steam coating at 433 K using water as a steam source [37]. Thus, the Al_2_O_3_ phase was considered not to be formed in our steam coating process. Consequently, the crystalline Mg(OH)_2_, Mg-Al LDH, and AlOOH phases in the films could be formed through the chemical reactions of Equations (1)–(3).

### 3.4. Effect of Al Content in the Mg Alloys on the Crystal Phases Formed in the Film

**Figure 5:**
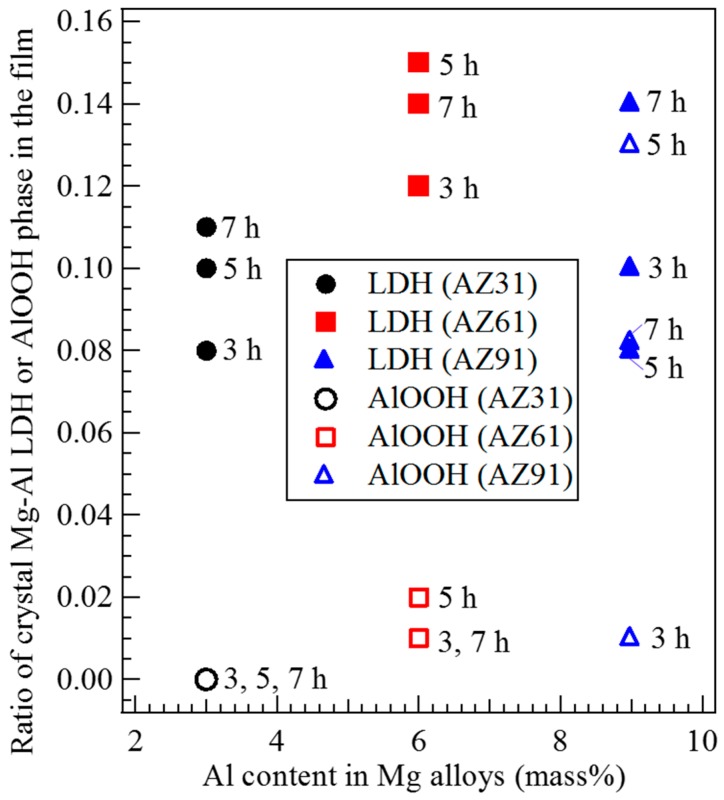
Relationship between the Al content in Mg alloys and the ratio of crystalline Mg-Al LDH or AlOOH phase formed in the films prepared at 423 K for 3 to 7 h.

The main crystal phase in all films prepared on AZ31, AZ61, and AZ91D Mg alloys by steam coating was Mg(OH)_2_ and minor crystal phases were found to be Mg-Al LDH and AlOOH as indicated in XRD patterns. Thus, the effects of the Al content in the Mg alloys on crystal phases formed in the film were investigated using the ratio of crystal phases formed in the film based on the peak intensity ratio. The relationship between the Al content in Mg alloys and the ratio of the crystalline Mg-Al LDH or AlOOH phase in the films prepared at 423 K for different treatment times is shown in Figure 5. The Mg-Al LDH content in the film prepared on AZ61 was relatively higher than those prepared on AZ31 and AZ91D. However, little notable difference in the Mg-Al LDH content in the film was observed between the different types of Mg alloys. However, the Mg-Al LDH content in the film prepared by steam coating could be controlled slightly. On the other hand, a notable difference in the AlOOH content in the film could be observed. The existing ratio of the crystalline AlOOH phase in the film increased with an increase in the Al content in the Mg alloys. However, the AlOOH ratio in the film prepared on AZ91D at 423 K for 3 h was small. This may be due to the difference in the dispersion states of Al atoms as a solid solution in the AZ91D Mg alloys. On the other hand, in the case of using AZ31 Mg alloy as the substrate, no AlOOH formation in the film was confirmed. This may be because the existing amount of the solid solution of Al atoms in the AZ31 Mg alloy was smaller than that for AZ61 and AZ91D. From these results, it can be speculated that the solid solution of Al atoms in Mg alloys could be utilized preferentially to form Mg-Al LDH because no AlOOH formation was observed in the film prepared on AZ31 despite the occurrence of Mg-Al LDH formation. This means that the formation of Mg-Al LDH may be thermodynamically more favorable than that of AlOOH in the film prepared by steam coating. 

### 3.5. Corrosion Resistance of AZ31, AZ61, and AZ91D Mg Alloys Coated with and without Films

**Figure 6:**
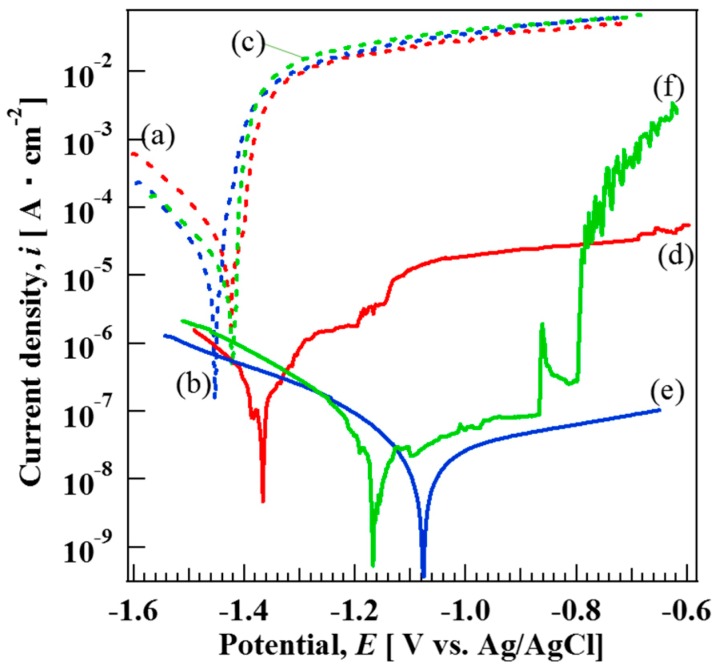
Potentiodynamic polarization curves of bare (a) AZ31, (b) AZ61, and (c) AZ91D and film-coated (d) AZ31, (e) AZ61, and (f) AZ91D in 5 mass% NaCl aqueous solution.

Figure 6 shows the polarization curves of the bare (a) AZ31, (b) AZ61, and (c) AZ91D Mg alloys and films prepared at 423 K for 5 h on (d) AZ31, (e) AZ61, and (f) AZ91D Mg alloys in 5 mass% NaCl aqueous solution. The corrosion potentials (E_corr_) of the bare AZ31, AZ61, and AZ91D in 5 mass% NaCl aqeuous solution are listed in Table 3. On the other hand, the corrosion current density (i_corr_) for the bare AZ31, AZ61, and AZ91D could not be estimated because of the absence in the anodic Tafel region. The E_corr_ values of the bare AZ31, AZ61, and AZ91D in 5 mass% NaCl aqeuous solution were estimated to be −1.42 V, −1.45 V, and −1.42V, respectively. In the cathodic region of the polarization curves for the three bare Mg alloys, hydrogen evolution dominates at more negative potentials than E_corr_, leading to an increase in the cathodic current densities. The anodic regions of the polarization curves for the three bare Mg alloys can be divided into two regions: (i) The abrupt increase in the current density from the potential in the range of −1.40 to −1.45 V (at low anodic overpotential) can be considered as the first region. In this region, the dominant dissolution reaction of Mg to Mg^2+^ ions occurred [38]. (ii) For more positive potential values, a current plateau can be observed as the second region, where the electrode surface was covered with a film identified as Mg(OH)_2_ [39]. The i_corr_ and E_corr_ values of the film coated AZ31, AZ61, and AZ91D were found to be 1.80 × 10^−7^ A cm^−2^ and −1.36 V, 2.37 × 10^−8^ A cm^−2^ and −1.07 V, and 1.39 × 10^−8^ A cm^−2^ and −1.13 V, respectively. These results indicate that all film coated Mg alloys had more positive corrosion potentials and lower current densities compared to those of bare Mg alloys, indicating a decrease of the cathodic and anodic active reactive sites on the sample surfaces. The positive shift of E_corr_ to more positive values could possibly be due to the change in the ratio between anodic and cathodic sites on the film surface. The corrosion potential is defined as a potential where the rate of anodic reactions is equal to that of cathodic reactions. Thus, E_corr_ is controlled by the kinetics or reactions occurring at the surface [40]. The current densities in the anodic and cathodic regions for all film-coated samples, that is, curve (d)–(f), were suppressed by the film formation, indicating that the films would be effective for improving the corrosion resistance of the three types of Mg alloys. On curve (d), the slight increase in the current density at approximately −1.20 and −1.14 V can be observed. The slight increase in the current density might be due to the existence of some cracks that formed in the film as shown in Figure 4a. The film coated AZ91D showed relatively low i_corr_ values among all samples. However, on the polarization curve (f) of the film coated AZ91D, an abrupt increase and decrease in the current density at approximately −0.86 V can be clearly observed. The increase and decrease in the current density on curve (f) might be due to the formation and dissolution of Mg(OH)_2_. In addition, the abrupt increase in current density at −0.78 V on curve (f) can also be detected, meaning that pitting corrosion occurred on the film prepared on AZ91D. Thus, the denseness and thickness of the film prepared on AZ91D was not high enough to protect against the corrosion reaction. On the other hand, curve (e), corresponding to the film prepared on the AZ61, showed complete passive behavior within the potential range of −1.0 to −0.64 V. In addition, curve (e) showed the lowest i_corr_ value among all samples. This indicates that the film had high corrosion resistance against Cl^−^ ions, indicating the film had superior corrosion resistance. The film prepared on the AZ61 had the highest ratio of the Mg-Al LDH content in the film among all samples. It has been reported that LDHs were effective materials to improve the corrosion resistance due to anion exchangeability [32]. Thus, the LDH content in the film might be related to the corrosion resistance of the film. However, the anion exchangeability of the LDH was not a dominant factor for improving the corrosion resistance of the film, although it may slightly contribute to the improvement of the corrosion resistance. The film thickness and denseness may be important factors for improving the corrosion resistance of the film. Our steam coating process enables an increase of the film thickness by controlling the process conditions. In addition, it can make the film denser by including the two or three phases, that is, Mg(OH)_2_, Mg-Al LDH, and AlOOH, with different sizes. The thickness and denseness of the film are generally important factors for improving the corrosion resistance because they can prevent a metal surface from contacting with a corrosive medium. All films prepared at 423 K for 5 h by the steam coating process were thick and dense as shown in Figure 4, so the films showed good corrosion resistance. It should be noted that the corrosion current densities for the films prepared on AZ61 and AZ91D were lower than that prepared on AZ31, indicating a decrease in the corrosion rate because the current density is proportional to the corrosion rate. From the XRD patterns, all films prepared on AZ61 and AZ91D were composed of Mg(OH)_2_, AlOOH, and Mg-Al LDH, whereas all films prepared on AZ31 were comprised of Mg(OH)_2_ and Mg-Al LDH. The film-coated AZ91D showed the highest ratio of AlOOH content in the film among all the samples. This may because the AZ91D Mg alloy includes a large amount of Al atoms.

**Figure 7:**
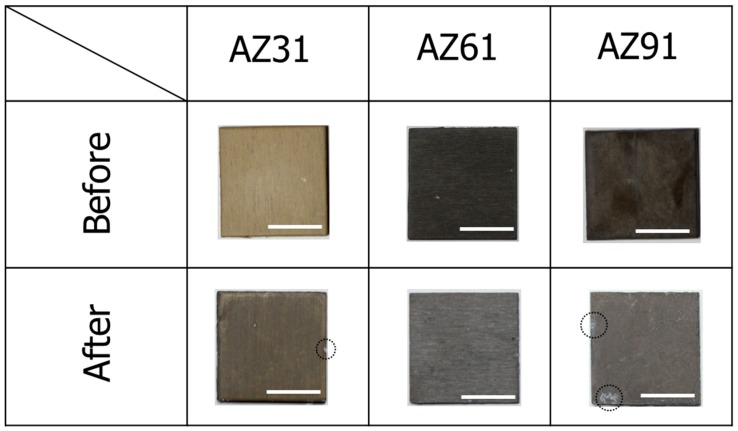
Appearances of films prepared at 423 K for 5 h on AZ31, AZ61, and AZ91D before and after immersion for 480 h in 5 mass% NaCl aqueous solution. All scale bars are 1 cm.

From the results of the XRD and polarization curve measurements, it can be considered that the formation of AlOOH in the film prepared by steam coating might lead to an improvement of the film denseness, resulting in an improvement of the corrosion resistance. The durability of the AZ31, AZ61, and AZ91D Mg alloys coated with films were further investigated by the immersion tests in 5 mass% NaCl aqueous solution for 480 h. The appearances of the AZ31, AZ61, and AZ91D Mg alloys coated with films before and after the immersion for 480 h are shown in Figure 7. The color of all film surfaces appeared to change slightly after the immersion. 

**Figure 8:**
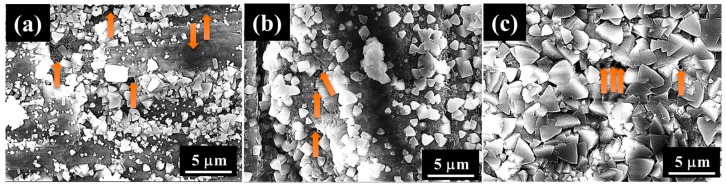
SEM images of films prepared at 423 K for 5 h on (**a**) AZ31, (**b**) AZ61, and (**c**) AZ91D after the immersion for 480 h in 5 mass% NaCl aqueous solution. All arrows show traces of pitting.

From their images, surface corrosion can be observed after the immersion. In the case of AZ31, the immersion in 5 mass% NaCl aqueous solution for 480 h resulted in slightly local dissolution at the middle of the right side of the film (denoted as a dotted circle line in Figure 7). The formation of corrosion products showing a gray color can be observed on the film coated AZ91D after the immersion in 5 mass% NaCl aqueous solution for 480 h (denoted as a dotted circle line in Figure 7). On the other hand, no physical damage in the film prepared on AZ61 can be observed, meaning that the film prepared on AZ61 had superior durability against 5 mass% NaCl aqueous solution. To investigate further changes in the surface states, SEM observation was performed. SEM images of the AZ31, AZ61, and AZ91D Mg alloys coated with films after immersion for 480 h are shown in Figure 8. The formation of the angulated substances was observed on all surfaces. No angulated substances could be detected on all surfaces before the immersion test in 5 mass% NaCl aqueous solution. Thus, the angulated substances were considered to be formed as corrosion products. From the SEM-EDS analysis, the atomic concentrations of C, O, and Mg after immersion for 480 h were found to be 3.0, 65.3, and 30.5 at.% for the film on AZ31, 4.5, 64.5, and 28.0 at.% for the film on AZ61, and 3.0, 65.5, and 31.0 at.% for the film on AZ91D, respectively. These results show that the corrosion products are mainly Mg(OH)_2_. Some pits could be observed on each film surface. This pit could be formed due to H_2_ evolution, which occurred because of the corrosion reaction. It should be noted that the formation amounts of pits were different and the existence rates of pitting on the film prepared on AZ61 were lower than those on AZ31 and AZ91D. This indicates that the durability against 5 mass% NaCl aqueous solution may change depending on the characteristics of the prepared films; that is, the film thickness and denseness. From these results, it can be concluded that the films prepared at 423 K for 5 h on the AZ61 Mg alloys showed the best corrosion resistance within the samples investigated.

## 4. Conclusions

Corrosion resistant films were prepared on three types of Mg alloys with different aluminum contents—that is, AZ31, AZ61, and AZ91D—by steam coating to investigate the relationship between the LDH content in the film and the Al content in the Mg alloys. SEM observation revealed that all films prepared on Mg alloys had compact and rough regions on the film. All films prepared on AZ61 and AZ91D were composed of crystalline Mg(OH)_2_, AlOOH, and Mg-Al LDH, whereas all films prepared on AZ31 were comprised of crystalline Mg(OH)_2_ and Mg-Al LDH as determined by XRD analysis. The Mg-Al LDH content in the film prepared on AZ61 was relatively higher than those prepared on AZ31. The ratio of the crystalline AlOOH phase in the film increased with an increase in the Al content in the Mg alloys. The film thickness changed depending on the treatment time and type of Mg alloy. Polarization curve measurements in 5 mass% NaCl solution demonstrated that the film prepared on the AZ61 showed complete passive behavior within the potential range of −1.0 to −0.64 V. In addition, immersion tests in 5 mass% NaCl aqueous solution for 480 h revealed that the film prepared on the AZ61 had superior durability against 5 mass% NaCl aqueous solution. These results indicated that the film prepared on AZ61 had the most superior corrosion resistance among all samples. We believe that the development of the steam coating process described here will greatly improve the corrosion resistance of a wide range of Mg alloys with further future work.

## Figures and Tables

**Table 1:** Peak intensity ratio of 011 reflection of Mg(OH)_2_, *I*_Mg(OH)2-001_, 003 reflection of Mg-Al LDH, *I*_LDH-003_, and 020 reflection of AlOOH, *I*_AlOOH-020_ to total peak intensities; that is, *I*_Mg(OH)2-0__11_ + *I*_LDH-003_ + *I*_AlOOH-020_, obtained from XRD patterns of the films prepared at 423 K for 5 h on AZ31, AZ61, and AZ91D Mg alloys.

Types of Mg Alloys	*I*_Mg(OH)2-001_	*I*_LDH-003_	*I*_AlOOH-020_
AZ31	0.90	0.10	0
AZ61	0.83	0.15	0.02
AZ91D	0.79	0.08	0.13

**Table 2:** Film thickness obtained from cross sectional SEM images of the films prepared at 423 K for different treatment times on AZ31, AZ61, and AZ91D Mg alloys (unit: µm).

Types of Mg Alloys	3 h	5 h	7 h
AZ31	5.7	37.8	44.2
AZ61	4.8	20.0	38.5
AZ91D	4.8	11.9	26.9

**Table 3:** Corrosion potential (*E*_corr_) and corrosion current density (*i*_corr_) of the films prepared at 423 K for 5 h on AZ31, AZ61, and AZ91D Mg alloys and bare AZ31, AZ61, and AZ91D Mg alloys obtained by Tafel extrapolation.

Types of Mg Alloys	Corrosion Potential, *E*_corr_ (V)	Corrosion Current Density, *i*_corr_ (A cm^−2^)
(a) AZ31 bare	−1.42	-
(b) AZ61 bare	−1.45	-
(c) AZ91D bare	−1.42	-
(d) AZ31	−1.36	1.80 × 10^−7^
(e) AZ61	−1.07	2.37 × 10^−8^
(f) AZ91D	−1.15	2.97 × 10^−8^
